# An Actuarial Pricing Method for Air Quality Index Options

**DOI:** 10.3390/ijerph16244882

**Published:** 2019-12-04

**Authors:** Zhuoxin Liu, Laijun Zhao, Chenchen Wang, Yong Yang, Jian Xue, Xin Bo, Deqiang Li, Dengguo Liu

**Affiliations:** 1 School of Economics and Management, Shaanxi University of Science and Technology, Xi’an 710021, China; liuzx0624@163.com (Z.L.); wangchenchen_sust@163.com (C.W.); ldqhb@126.com (D.L.); 2 China Institute for Urban Governance, Shanghai Jiao Tong University, Shanghai 200030, China; 3 Sino-US Global Logistics Institute, Shanghai Jiao Tong University, Shanghai 200030, China; 4 School of Arts and Sciences, Shanxi University of Science & Technology, Xi’an 710021, China; yangyong@sust.edu.cn; 5 Appraisal Center for Environment and Engineering, Ministry of Environmental Protection, Beijing 100012, China; boxinet@gmail.com; 6 School of Automotive Studies, Tongji University, Shanghai 201804, China; ldg@sheemc.cn; 7 Shanghai Environment Monitoring Center, Shanghai 200235, China

**Keywords:** air quality index, options, Ornstein–Uhlenbeck model, actuarial pricing, risk hedging

## Abstract

Poor air quality has a negative impact on social life and economic production activities. Using financial derivatives to hedge risks is one of the important methods. Air quality index (AQI) options are designed to help enterprises cope with the operational risk caused by air pollution. First, the expanded Ornstein–Uhlenbeck model is established using an autoregressive-generalized autoregressive conditional heteroscedasticity (AR-GARCH) method to predict AQI for a city. Next, the average AQI is constructed to be as the underlying index for the AQI options. We then priced AQI options using an actuarial method with an Esscher transform. Meanwhile payoff functions for the options are established to let enterprises hedge against the operational risk caused by air pollution. Finally, we determined the price of AQI options using data from Xi’an, China, and the example of a tourism enterprise as a case study of how AQI options can be applied to hedge against operational risk for enterprises. With AQI options trading, enterprises can hedge against operational risks caused by air pollution. The applicability of AQI options is wide, it can also be applied in other cities or regions.

## 1. Introduction

Pollution is becoming an increasingly serious problem, especially in developing countries. The prevention and control of air pollution has therefore become one of the most important issues faced by countries around the world. On the one hand, air quality affects our health and daily life, while on the other hand, it also profoundly affects economic production activities. Days with high levels of air pollution will damage the human respiratory system and increase the incidence of cardiovascular diseases [1]. This increases medical expenditures. A Cornell University study showed that for every 10 µg/m^3^ decrease in the concentration of particles smaller than 2.5 µm (PM_2.5_) in China, national medical expenditures would decrease by US$42 billion, which amounted to 7% of China’s total medical expenditure in 2015 [2]. Air pollution can also decrease well-being, since it forces us to reduce outdoor activities on smoggy days and increase spending on protective equipment, such as masks and air purifiers.

Air pollution also has a negative impact on economic production activities. For example, agriculture, transportation, tourism, and other industries suffer from air pollution. Many air pollutants decrease crop growth, leading to significant economic losses [3,4]. Poor air quality affects visibility and poses a threat to transportation operations and safety [5]. The deterioration of environmental quality also damages the image of tourist destinations [6], thereby reducing their attractiveness to tourists, and consequently reducing tourism revenues. The impact of air pollution on business operations is similar to that of weather risks such as temperature and rainfall. People and enterprises both take measures to hedge against the risk created by different forms of extreme weather. For catastrophic weather events, such as tsunamis and severe rainstorms, which cause serious property losses and high casualties, insurance can be used to shift the risk from individuals and enterprises to insurance companies [7,8]. For non-catastrophic weather fluctuations, such as high or low temperatures, rainfall, and other frequent events, the losses are relatively small, but fluctuations in these weather conditions may still have considerable impacts on enterprises that are sensitive to weather. 

Using derivatives to hedge against the risks created by weather factors can reduce fluctuations in earnings and help a company hedge against risk. Weather derivatives have been widely used to manage weather risks such as extreme temperatures and precipitation. The Chicago Mercantile Exchange has introduced the use of these weather risks as the underlying indexes. Inspired by these weather derivatives, we designed the present study to explore the use of a particular financial derivative (options) to determine its potential to help enterprises hedge against the operational risks caused by air pollution.

The structure of the rest of this paper is organized as follows. Section 2 provides the literature review. In Section 3, an expanded Ornstein–Uhlenbeck (O–U) forecasting model using the autoregressive-generalized autoregressive conditional heteroscedasticity (AR-GARCH) method is established, and then an average air quality index (AAQI) is constructed for use as the underlying index for the AQI options. Next, we develop the actuarial pricing equation for AQI options using the Esscher transform method, and illustrate the basic principle for hedging against risk using options. Using Xi’an, China, as a case study, we analyze the pricing results for AQI options and illustrate their use for a typical tourism enterprise in Section 4, and then summarize our conclusions in Section 5.

## 2. Literature Review

Air pollution has attracted increasing attention around the world. Several researchers have studied the comparability of air pollutant indexes and the assessment of air quality [9,10]. Suggestions and countermeasures have been proposed from the perspectives of pollution control and governance [11,12]. In addition, governments in different countries have also taken a series of measures to reduce pollution, such as introducing policies and regulations, the Clean Air Acts of the UK and US, the federal pollution control law of Germany, and the Law of the Prevention and Control of Atmospheric Pollution of China [13,14]. Provincial governments in many Chinese cities have also enacted policies to reduce air pollution, such as the city of Xi’an, which imposed traffic restrictions on motor vehicles [15,16].

Many researchers have studied the use of financial means to manage weather risks [17,18,19]. These include insurance, and weather derivatives based on futures, options, and other financial instruments. Insurance is widely used in agriculture to protect farmers against crop losses. Due to ethical problems such as adverse selection (i.e., due to information asymmetry) in traditional agricultural insurance [20], recent research has mainly focused on weather index insurance [21,22]. However, insurance is mostly used for catastrophic events, whereas derivatives provide a more flexible risk management tool for non-catastrophic weather risks [23,24]. Gao et al. [25] found that using options as a hedging strategy can help retailers improve their expected profits. Hardle and Osipenko [26] used Leipzig, Germany as an example to illustrate the effectiveness of hedging against risks using temperature futures. Moon and Son [27] took the natural gas industry as an example to illustrate the advantages of using weather derivatives and the requirements for using such derivatives. Hainaut [28] used two weather derivatives (futures and options) based on a cumulative average temperature index to hedge against temperature risks.

The Chicago Mercantile Exchange launched temperature index futures in 1999, and since then, weather derivatives have developed rapidly and now represent a mature international trading market [29,30]. Compared with futures, options offer a price discovery function that makes the trading price more realistic than futures prices [31]. Options give the buyer the right to choose, thereby making the trading more flexible.

Research on temperature derivatives has mainly attempted to predict the underlying index based on the O–U model, which can capture the characteristics of seasonality, reversion to the mean, and stochastic properties of the changes in temperature and in other time series [32,33,34,35]. Other methods have been used to model temperature series, such as autoregressive moving-average and conditional autoregressive models. Li et al. [36] expanded the O–U model by using an AR-GARCH model to optimize the fitting accuracy for the prediction of average daily temperatures in Shanghai, China. However, in subsequent research on using the O–U model to predict air pollutants and air quality, researchers sometimes incorrectly assumed that the randomly perturbed variable followed a normal distribution [37,38]. In addition, these authors did not test for autocorrelation and conditional heteroscedasticity; both problems must be tested for and, if present, must be solved to preserve the accuracy of predictions.

The key to successful use of options lies in construction and pricing of the underlying index. Most research falls into one of two categories. Temperature derivatives, which are mainly based on heating and cooling degree-days, account for the largest proportion of weather derivatives [39]. The other category of underlying indexes for weather derivatives includes precipitation, snowfall, and frost. However, few researchers have studied derivatives with air pollutants as the underlying index. Li and Zhu [37] designed an options contract based on the haze index, with the PM_2.5_ concentration used to quantify haze, and took Beijing as an example to price and analyze the options. This was an innovative index for derivatives. However, the PM_2.5_ concentration cannot fully reflect the severity of air pollution, so its application may be limited. Xue et al. [38] designed an air pollution options contract based on a multi-pollutant air quality index (AQI), further expanding the available indexes suitable for use in derivatives. The design of an underlying index based on AQI is more comprehensive than a single-pollutant index, but it may not describe the actual situation sufficiently well, and must therefore be improved.

The traditional non-arbitrage pricing represented by the model of Black and Scholes [40] is most widely used in options pricing, but it is not applicable to weather derivatives because the market for weather derivatives is incomplete; that is, the underlying index cannot be traded [41,42]. Jewson and Brix [43] noted that weather options pricing combines actuarial techniques with market-based comprehensive pricing, and emphasizes actuarial pricing in most cases. Market-based pricing needs to be based on the current market price by extracting the implied market price of risk. However, due to the absence of actual market transaction data, the market price of risk cannot be calculated. Since Bladt and Rydberg [44] proposed the use of actuarial pricing, this method has been widely used in options pricing [45]. As an important classical tool in actuarial pricing, the Esscher transform is widely used in options pricing and has a wide range of applications [46].

To solve the research problems described in this literature review, we established an expanded O–U model using the AR-GARCH method to eliminate autocorrelation and conditional heteroscedasticity from the residuals, thereby letting us predict AQI more reasonably. To make the underlying index of AQI options more realistic, we developed AAQI, and we priced AQI options using the actuarial method with an Esscher transform.

## 3. Materials and Methods

### 3.1. Expanded O–U Model Using the AR-GARCH Method

Since AQI exhibits variations similar to those of temperature, which can be simulated using O–U models, we chose an expanded O–U model to simulate variation in the AQI time series. The O–U model is expressed by a stochastic differential equation in the following form [47]:

(1) dD(t)=ds(t)+φ[D(t)−s(t)]dt+σ(t)dW(t)

where D(t) represents the AQI values on day t, s(t) is a bounded and continuously differentiable deterministic function for describing the trends and seasonal variations of the AQI time series, and D(t)−s(t) indicates that AQI excludes trends and seasonal variations. φ is the rate at which AQI returns to its mean level after a large change. σ(t) is the daily volatility of AQI, and dW(t) indicates a Wiener process. Taking days as the units of measurement, Equation (1) can be discretized into:

(2) ΔD(t)=Δs(t)+φ[(D(t−1)−s(t−1)]Δt+σ(t−1)ΔW(t)

where Δt=1, ΔD=D(t)−D(t−1), Δs=s(t)−s(t−1), ΔW=W(t)−W(t−1), and ΔW is N(0,1), This equation can be further simplified into:

D(t)−s(t)=(1+σ)[D(t−1)−s(t−1)]+σ(t)ε(t), t=1,2,…365.

The O–U time series model can be summarized as follows:

(3) $$D_{t}=S_{t}+C_{t}+\sigma_{t}\varepsilon_{t}$$

where $D_{t}$ is the AQI on day t. $S_{t}$ accounts for the trends and seasonal variations of the AQI time series, and which represents the long-term average of AQI. $C_{t}$ is the periodicity of the AQI time series, and $\sigma_{t}$ is the daily volatility of AQI. $\varepsilon_{t}$ is a variable that accounts for random perturbation.

The O–U model is used to fit the parameters in Equation (3) in three steps. First, $S_{t}$ is estimated to account for the trends and seasonal variations in AQI. Second, $C_{t}$ is estimated by autoregression of the AQI time series with trends and seasonal variations excluded. Third, $\varepsilon_{t}$ is estimated.

#### 3.1.1. Trends and Seasonal Variations of the AQI Time Series

The Fourier series expansion of Equation (3) is expressed as follows:

(4) $$y=a+bt+a_{0}+\sum_{m=1}^{I_{1}}a_{m}\sin(mwt)+\sum_{n=1}^{J_{1}}b_{n}\cos(nwt)$$

where a+bt represents the trend for variation of the AQI time series, and the rest of the equation accounts for the seasonal variation.

#### 3.1.2. Periodicity of the AQI Time Series

The fitting of periodicity needs to exclude trends and seasonal variations, and the model for parameter estimation needs to be judged according to the characteristics of the autocorrelation coefficient and partial autocorrelation coefficient. Table 1 summarizes the optimal model selection.

#### 3.1.3. Variable to Describe Random Perturbation in the AQI Time Series

The original O–U model estimates the random disturbance term using a Fourier series expansion of the residual term:

(5) $$\sigma_{t}^{2}=c+\sum_{i=1}^{I_{1}}c_{i}\sin(iwt)+\sum_{j=1}^{J_{1}}d_{j}\cos(jwt)$$

However, the assumption that $\varepsilon_{t}$ follows a standardized normal distribution in Equation (3) is not realistic, and the problems of autocorrelation and conditional heteroscedasticity are not solved for the residual terms. Li et al. [36] first used an AR-GARCH model for temperature derivatives to solve the problems of autocorrelation and conditional heteroscedasticity of a randomly perturbed temperature variable. We therefore introduced the AR-GARCH model to expand the O–U model, so as to better meet the hypothesis of a standardized normal distribution and to improve the accuracy of the O–U model’s prediction.

The AR-GARCH model first accounts for autocorrelation of the residual terms and uses an autoregressive (AR) model to fit the residuals. The GARCH model is then used to fit the conditional heteroscedasticity of the residual terms. In the GARCH (p,q) model, p represents the lag of GARCH, and q represents the lag of autoregressive conditional heteroskedasticity (ARCH).

We adopted ARCH (1) and GARCH (1) for this model. After introducing the AR-GARCH (1,1) model, the variable that accounts for random perturbation can be rewritten as:

(6) $$\sigma_{t}^{2}=Y_{t}+\sum_{i=1}^{q}\beta_{i}(\sigma_{t-i}^{2}-Y_{t-i})+\gamma_{1}\sigma_{t-1}^{2}+\gamma_{2}\varepsilon_{t-1}^{2}$$

In Equation (6), $Y_{t}$ is the seasonal fluctuation of AQI’s random fluctuation term, q is the lag order of AR model, $\gamma_{1}$ and $\gamma_{2}$ are the coefficients of the GARCH (1,1) model, and $\varepsilon_{t-1}$ follows a standardized normal distribution with a mean of 0 and standard deviation of 1.

### 3.2. Index Construction for AQI Options

AQI is a daily-scale indicator of air quality that describes air quality by quantitatively evaluating the level of air pollution. Based on China’s national air quality standards and the impact of various pollutants on the ecological, environment and human health, China issued a new environmental air quality standard (GB3095-2012) in March 2012. The government monitors six pollutants in the standard: sulfur dioxide, nitrogen dioxide, carbon monoxide, ozone, particles smaller than 10 µm (PM_10_), and PM_2.5_. AQI equals the maximum index value for the six pollutants on a given day. AQI ranges from 0 to 500 and is divided into six grades (Appendix A).

The construction of the underlying index is an important step in the design of derivatives. Xue et al. [38] did not differentiate among the different levels of pollution when they designed their average deviation index (ADI) for air pollution options, resulting in an unrealistic index design. For example, the average deviation index can be low when AQI is high, and this is clearly inconsistent with the actual situation. Therefore, we redesigned the underlying index for AQI-based options to make it more realistic and thus, more representative of the actual situation.

We defined a new index, AAQI, which represents the average value of AQI over a given period of time. Based on the AQI classification criteria in Appendix A, we chose the lower limit of each AQI level as the threshold. Depending on the desired duration of the options contract, AAQI can be based on durations such as 1 week, 1 month, or 1 quarter, thereby providing considerable flexibility in defining the terms of the contract. *AAQI* is expressed as follows:

(7) $$AAQI=\frac{1}{n}\sum_{i=1}^{n}\left(l_{j}+\frac{AQI_{i}-L_{A}}{L_{A}}\right)$$

where

(8) $$l_{j}=\left\{ \begin{array}{l} 1,\;\;\;\;51\leq AQI_{i}\leq 100\\ 2,\;\;\;\;101\leq AQI_{i}\leq 150\\ 3,\;\;\;\;151\leq AQI_{i}\leq 200\\ 4,\;\;\;\;201\leq AQI_{i}\leq 300\\ 5,\;\;\;\;\;AQI_{i}\geq 301 \end{array}\right.;\;L_{A}=\left\{ \begin{array}{l} 51,\;\;\;\;51\leq AQI_{i}\leq 100\\ 101,\;\;\;\;101\leq AQI_{i}\leq 150\\ 151,\;\;\;\;151\leq AQI_{i}\leq 200\\ 201,\;\;\;\;201\leq AQI_{i}\leq 300\\ 301,\;\;\;\;\;AQI_{i}\geq 301 \end{array}\right.$$

where $AQI_{i}$ represents AQI on day *i*, and *n* represents the duration of the AQI options contract. $L_{A}$ is the lower limit of each AQI level. A higher AQI indicates more serious pollution and produces a higher AAQI. To distinguish the pollution degree at each level, $l_{j}$ (a constant that represents the AQI level) is added in the calculation of AAQI to improve the realism of the index. Since there are few cases when AQI value is lower than 51, we ensured that the index calculation would be meaningful (i.e., we ensured that AQI is < 51 would not lead to the use of $L_{A}$ as 0 in the calculation) and improved the pricing accuracy by treating AQI values below 51 as having $L_{A}=51$.

### 3.3. Actuarial Pricing for AQI Options Based on the Esscher Transform

If we assumed that *AAQI* is D(t) at time t and X(t) is the growth rate of the underlying index, then:

(9) D(t)=D(0)exp[X(t)], t≥0

If we take [0,T] as the duration of the options contract, T as the expiration date, and t as the pricing date, then the growth index at the expiration of the AQI options contract can be expressed as:

(10) X(T)=X(t)A(T−t)

The *AAQI* at the expiration of the contract is as follows:

(11) D(T)=D(t)exp[A(T−t)]

If we assumed that H is the maximum delivery price and K is the options strike price (i.e., the price at which the options can be exercised), then using call options as an example, the payoff function P(T) for the options is as follows:

(12) $$P(T)=\left\{ \begin{array}{l} 0,\;\;\;\;D(T)<K\\ D(T)-K,\;\;K\leq D(T)<H\\ H-K,\;\;\;\;D(T)>H \end{array}\right.$$

The Esscher transform has been proven to represent the unique equivalent martingale measure, in which the conditional expectation in a sequence of random variables equals the present value [48]. Under the equivalent martingale measure, the price of a financial asset equals the discounted present value of future returns.

If we used “*” to indicate the value after the Esscher transform, then the options price based on AQI can be obtained by directly discounting the expectation yield (i.e., the yield at the risk-free discount rate) at the expiration of the options contract. This highlights the superiority of the Esscher transform. Based on these basic principles, *AAQI* at time t can be expressed as:

(13) $$D(T)=\exp[-r(T-t)]E_{t}^{*}[D(t)\exp A(T-t)]$$

This can be simplified as follows:

(14) $$\exp[r(T-t)]=E_{t}^{*}[\exp A(T-t)]$$

After Esscher-transformation of the incremental process A(T−t) using the parameter $h^{*}$, we can obtain its moment-generating function, $M[z,T-t;h^{*}]$. When z=1, the equation can be expressed as:

(15) $$\exp[r(T-t)]=M_{A}[1,T-t;h^{*}]$$

For ease of calculation, we assumed that T−t=u, where u∈[0,T]. In a random process after Esscher transforms of the parameter, F(x,t;h) and f(x,t;h) are the cumulative probability-distribution function and probability-density function, respectively. At this time, the price of call options is:

(16) $$\begin{array}{l} C_{t}=\exp[-r(T-t)]E_{t}^{*}[P(T)]\\ \;\;\;\;\;\;\;\;\;\;\;\;\;\;=\exp[-r(u)]\{\int_{K}^{H}[(D(T)-K)f_{X}(x,T-t;h^{*})]dx+\int_{H}^{+\infty }[(H-K)f_{X}(x,T-t;h^{*})]dx\} \end{array}$$

If we assume m=ln[H/D(t)] and n=ln[K/D(t)], then Equation (16) can be simplified as follows:

(17) $$\begin{array}{l} C_{t}=\exp(-r(u))\int_{n}^{m}[(D(T)-K)f_{X}(x,T-t;h^{*})]dx+\\ \;\;\;\;\;\;\;\;\;\;\;\;\;\;\;\;\;\;\;\;\;\;\exp(-r(u))\int_{m}^{+\infty }[(H-K)f_{X}(x,T-t;h^{*})]dx \end{array}$$

The call options price for AQI can be summarized as follows:

(18) $$\begin{array}{l} C_{t}\;=D(t)[F(m,T-t;h^{*}+1)-F(n,T-t;h^{*}+1)]\\ \;\;\;\;\;\;\;\;\;\;\;\;\;\;\;\;\;\;\;\;\;-K\exp(-r(T-t))[F(m,T-t;h^{*})-F(n,T-t;h^{*})]\\ \;\;\;\;\;\;\;\;\;\;\;\;\;\;\;\;\;\;\;\;\;+(H-K)\exp(-r(T-t))[\;1-F(m,T-t;h^{*})] \end{array}$$

Appendix B provides details of the calculation leading to this equation. The calculation of put options is similar to that for call options.

The economic implications expressed in Equation (18) are as follows:

$\left[F(m,T-t;h^{*}+1)-F(n,T-t;h^{*}+1)\right]$ represents the probability under the new probability measure that has been Esscher-transformed with parameter $\left(h^{*}+1\right)$, when AAQI is greater than the strike price K and less than the maximum delivery price H.

$\left[F(m,T-t;h^{*})-F(n,T-t;h^{*})\right]$ represents the probability that under the Esscher transform using the $h^{*}$ parameter, AAQI is greater than the strike price K and less than the maximum delivery price H.

$\left(H-K)\exp(-r(T-t))[1-F(m,T-t;h^{*})\right]$ means that the return is fixed as (H−K) when AAQI exceeds the maximum delivery price, and the relative risk neutral probability is $\left[1-F(m,T-t;h^{*})\right]$.

The specific pricing equation needs to identify the type of distribution of the incremental process A(T−t), which needs to be determined according to the results of subsequent empirical analysis.

### 3.4. Payoff Function for AQI Options

**Figure 1:**
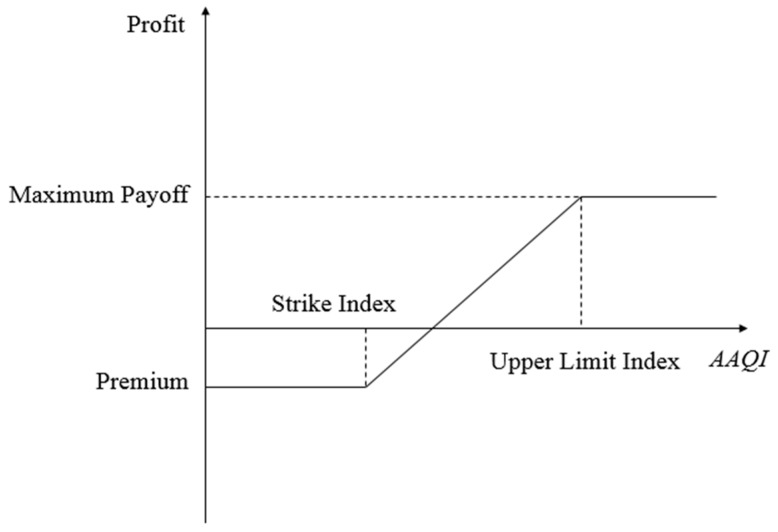
Illustration of air quality index (AQI) call options.

Realistic pricing of AQI options can help enterprises to lock in the range of risk caused by air pollution in advance, determine the cost of hedging against this risk, and thereby better manage their operational risk. AQI options give the contract holder the right to execute the contract or to not execute the contract. When the AAQI at the expiration of the contract exceeds the strike index, the enterprise can choose to execute the options contract. If AAQI does not exceed the strike index, the enterprise will not execute the contract and will only lose the premium paid to acquire the options. Figure 1 shows the profit and loss chart for purchasing AQI call options.

If we assumed that D(T) represents the AAQI at the expiration of the contract, and m represents the tick size (i.e., the price per unit of AAQI). The strike index is K, and H is the maximum delivery index for AQI. (H−K) then represents the maximum payoff. From the perspective of the buyer, the payoff function P(T) for call options can be expressed as:

(19) $$P(T)=\left\{ \begin{array}{l} 0,\;\;\;\;\;\;\;\;D(T)\leq K\\ m[D(T)-K],\;\;\;K<D(T)<H\\ m(H-K),\;\;\;\;\;\;D(T)\geq H \end{array}\right..$$

**Figure 2:**
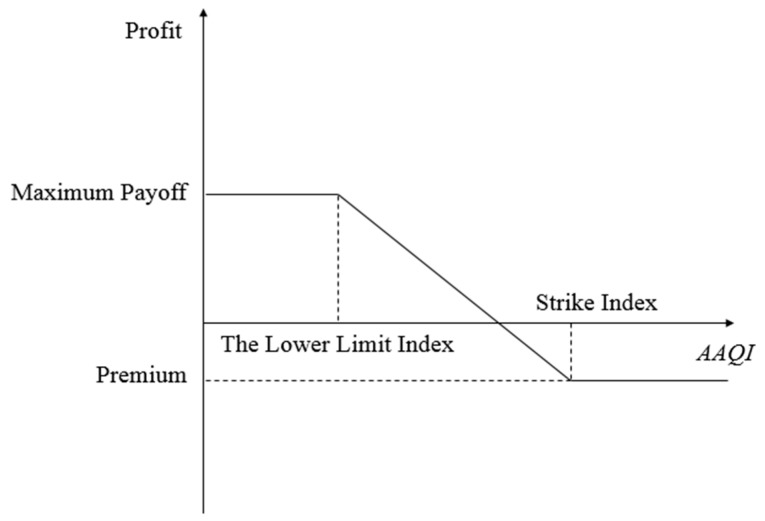
Illustration of AQI put options.

In put options, if AAQI is lower than the strike index, the company will execute the contract, whereas if AAQI is higher, it will not execute the contract. Put options are generally used to account for the adverse impact of low AQI on earnings, such as in the case of an enterprise that manufactures air purifiers. Figure 2 shows the profit and loss for put options.

From the buyer’s point of view, the payoff function for put options can be expressed as:

(20) $$P(T)=\left\{ \begin{array}{l} m(K-H),\;\;D(T)\leq H\\ m(K-D(T)),\;\;\;H<D(T)<K\\ 0,\;\;\;\;\;\;\;\;\;D(T)\geq K\;\;\; \end{array}\right..$$

In practice, the buyer must choose an amount and type of AQI options to be purchased according to the range of risk, so the payoff amount will vary according to the situation.

## 4. Case Study

**Figure 3:**
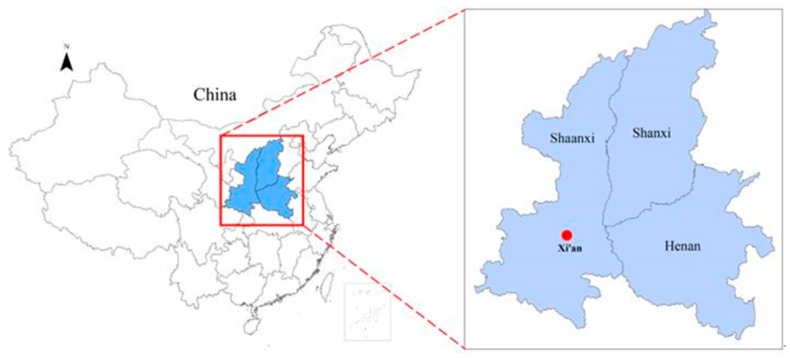
The location of Xi’an in China.

Xi’an (34°15′ N, 108°56′ E; Figure 3), the capital city of China’s Shaanxi province, is currently one of the most polluted cities in China according to the notice issued by the Ministry of Ecology and Environment of the China and is therefore a key city for pollution control [49]. To demonstrate the use of the method developed in Section 3, we used Xi’an’s AQI values from 2013 to 2018. We developed a prediction equation for AQI using data from 2013 to 2017 as the sample, and used data from 2018 to validate the developed model.

### 4.1. Parameter Estimation for the Expanded O–U Model Using AR-GARCH

**Figure 4:**
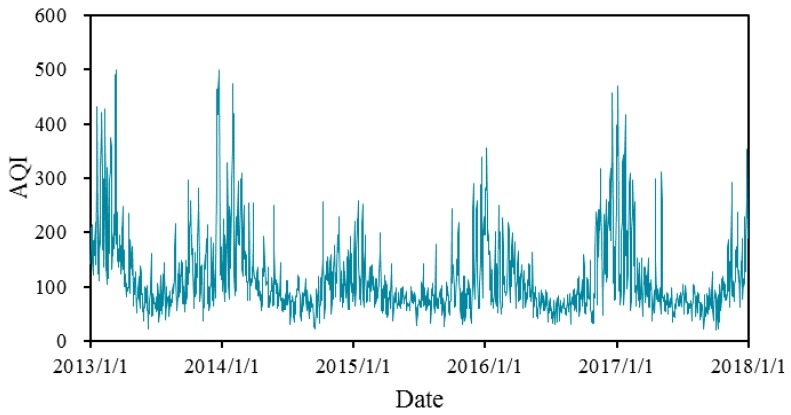
The tendency chart of AQI in Xi’an.

Figure 4 shows the trends of AQI from 2013 to 2017 in Xi’an. The graph shows that AQI has obvious seasonal trends and high variation within a season, with higher values in the first quarter (from January to March) and the fourth quarter (from October to December). Although the fluctuation is high, AQI varies around a mean value and shows the characteristic of reversion towards the mean. Based on this data series, we fitted the AQI trend in three steps, described in Section 4.1.1, Section 4.1.2 and Section 4.1.3.

#### 4.1.1. Trends and Seasonal Variation in the AQI Time Series

First, we estimated the parameters a and b for $S_{t}$ according to Equation (4), and used these values to represent the trend in the variation. After excluding trends in the variation from AQI, we then estimated the seasonal variation. The results are shown in Table 2.

**Figure 5:**
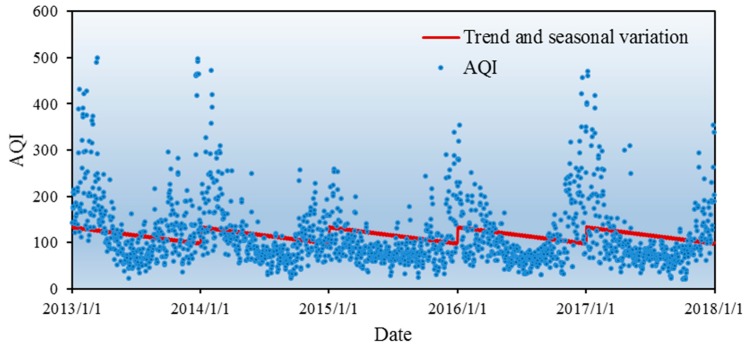
The Fourier-series fitting of St in Equation (4).

Table 2 shows that a=133.657 and b=−0.097, and the regression was statistically significant (*p* < 0.05). Since b was negative, this means that AQI decreased over time, indicating that air pollution control was effective, but limited. Figure 5 shows the results of the fitting. The seasonal variation was obvious. From the fourth quarter to the first quarter of the next year, AQI shows an upward trend in winter, whereas in the second quarter (April–June) and the third quarter (July–September), it shows a downward trend. This agrees with the real-life experience. 

#### 4.1.2. Periodicity of the AQI Time Series

**Figure 6:**
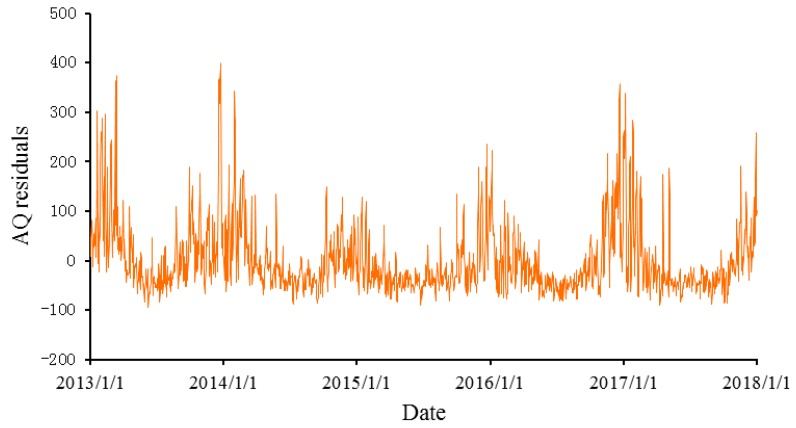
The trends for the AQI residuals.

**Figure 7:**
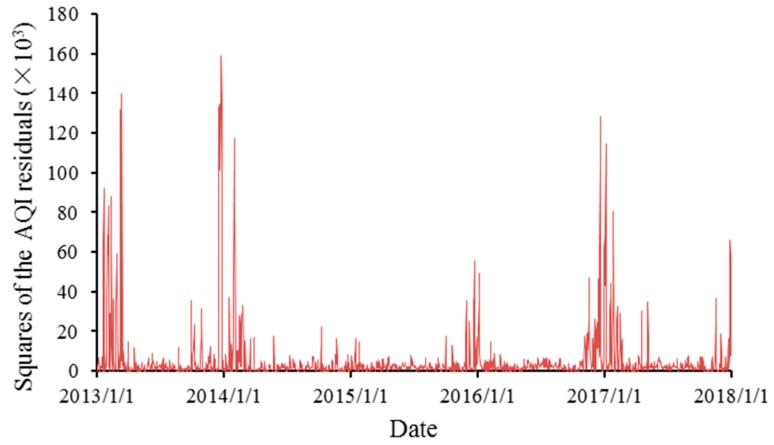
The trends for the squares of the AQI residuals.

Figure 6 and Figure 7, respectively, show the AQI residuals and the squares of the residuals with trends and seasonal variations excluded. The residuals show obvious fluctuations and clustering, indicating that a higher-order autocorrelation might exist, so further discussion is needed.

**Figure 8:**
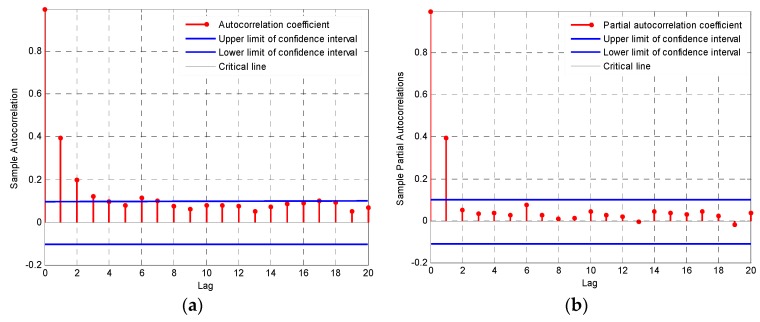
Autocorrelation graph (**a**) and partial autocorrelation graph (**b**) for the AQI residuals.

We analyzed periodicity in the AQI residuals after trends and seasonal variations were excluded, and obtained the autocorrelation coefficient and partial autocorrelation coefficient for the residuals (Figure 8). Figure 8 shows that the autocorrelation coefficient had a tail in the residual term, whereas the partial autocorrelation coefficient graph was censored at lag orders starting with 2. According to the model selection criteria in Table 1, the simple autoregressive model was most appropriate. We therefore selected model AR (2) to estimate the value of $C_{t}$.

$X_{t}$ represents the residuals of AQI after trends and seasonal variations have been excluded, and β is the parameter value that must be estimated. The results of the AR (2) model are as follows:

(21) $$X_{t}=0.376X_{t-1}+0.05X_{t-2}+\sigma_{t}\varepsilon_{t}.$$

#### 4.1.3. Variable that Accounts for Random Perturbation in the AQI Time Series

$Y_{t}$ in Equation (6) is expanded using a Fourier series, taking the expansion term as $I_{1}=J_{1}=5$ based on the 5 years of data used to develop our model. Table 3 shows the resulting parameter values.

If we excluded $Y_{t}$ from the AQI variable that accounts for random perturbation, the augmented Dickey–Fuller unit root test revealed a stationary sequence, with *t* = −17.157, versus a critical value of −3.968 at *p* < 0.01, and there was a first-order autocorrelation. Therefore, we established the AR (1) autoregression model (i.e., q=1 in Equation (6)), which can be expressed as:

(22) $$\sigma_{t}^{2}-Y_{t}=\beta_{1}(\sigma_{t-1}^{2}-Y_{t-1}).$$

The regression in Equation (22) shows that the residual has obvious clustering of volatility. A conditional heteroscedasticity test for the AR (1) model shows the results as shown in Table 4.

Thus, the original hypothesis was rejected, indicating the existence of conditional heteroscedasticity. The GARCH (1,1) model was used to re-estimate the autoregressive equation with β_1_ = 0.610, γ_1_ = 0.100, and γ_2_ = 0.836.

Th conditional heteroscedasticity test was performed on AR (1)-GARCH (1,1), and the results are shown in Table 5. Thus, none of the parameters was statistically significant, and the original hypothesis was accepted. The coefficients of ARCH and GARCH were both greater than 0, and the sum of the coefficients was 0.936, which was less than 1, and this satisfied the constraint conditions. These results show that the conditional heteroscedasticity of the residuals could be eliminated by using the GARCH (1,1) model. 

By successively fitting the trend variation, seasonal change, periodicity, and randomly perturbed variable of AQI, we developed a prediction model that could be used to predict AQI in 2018.

### 4.2. Pricing of AQI Options

Based on the predicted AQI values in 2018 described in Section 4.1, the incremental process of AAQI obeys the Wiener distribution, and on that basis, a specific options pricing formula can be determined (Appendix C).

**Figure 9:**
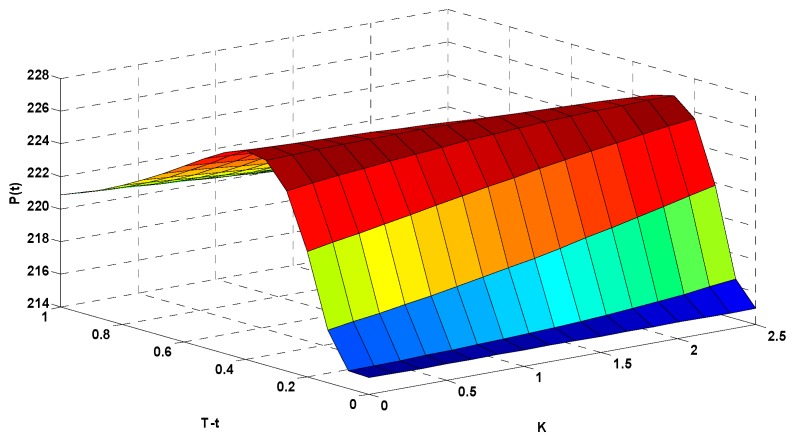
Results of the AQI options pricing calculation.

Due to the obvious seasonality of AQI in Xi’an, a quarterly options contract with a period of 3 months provides an example of options pricing. The air pollution in Xi’an was most serious in the first quarter, when AQI was highest. Therefore, we used the predicted AQI from 1 January 2018 to 31 March 2018 to price the options. According to Equation (7), AAQI was 2.28 from January to March 2018. The interest rate for 1-year Chinese treasury bonds in 2018 was taken as the risk-free rate (at 2.3%) in the options model. The AAQI at the upper limit of the maximum range of AQI within the contract duration was the maximum delivery index; that is, H=2.5. Figure 9 shows the pricing results for AQI call options.

**Figure 10:**
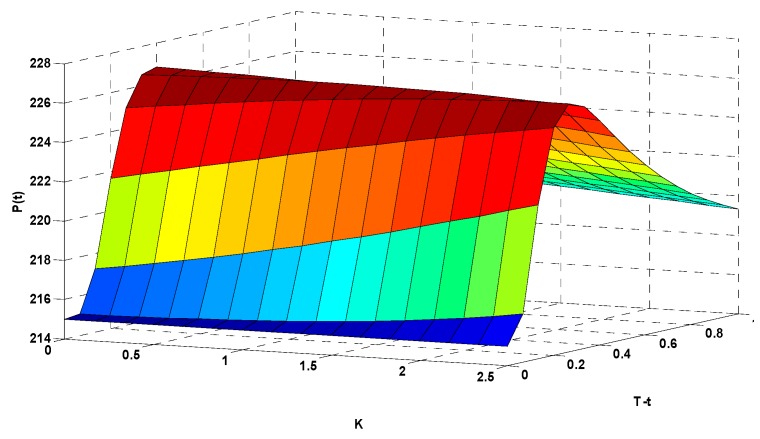
Results of the AQI options pricing calculation.

**Figure 11:**
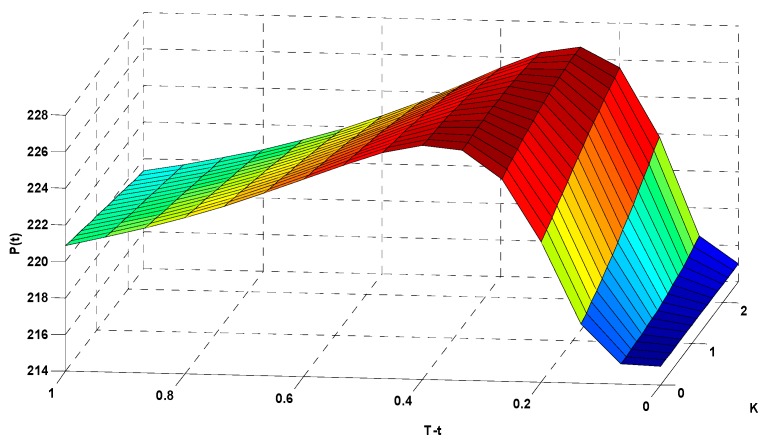
Results of the AQI options pricing calculation.

In Figure 9, the *x*-axis (K) represents the option’s strike index, the *y*-axis (T−t) represents the option’s expiration time, and the *z*-axis (P(t)) represents the corresponding options price (in CNY). To more clearly illustrate the relationships among the options strike index, expiration time, and corresponding options price, we rotated Figure 9 to show the data from different angles in Figure 10 and Figure 11.

This graph is the same as the graph in Figure 9, but shown from a different perspective.

Figure 10 emphasizes the relationship between the strike index and the options price. When the expiration time T−t was fixed at 0.6 (54 days before expiration of the options contract), different strike indexes correspond to different options prices. When K=0.5, P(t)=224.39 CNY. When K=1.0, P(t)=223.98 CNY. When K increased to 1.5, P(t)=223.55 CNY. When K=2.0, P(t)=223.09 CNY. This shows that as the strike index of the call options increased, the value of the call options decreased.

Figure 11 focuses on the relationship between the expiration time and the options price. When the strike index K was fixed at 2.0, the price of the options varied in response to changes in the duration. When T−t=0.8, P(t)=221.03 CNY. When T−t=0.6, P(t)=223.09 CNY. When T−t=0.4, P(t)=225.84 CNY. When T−t=0.2, P(t)=224.94 CNY. The results show that the options price increased with increasing expiration time, and decreased more gradually when approaching the expiration date.

This graph is the same as the graph in Figure 9, but shown from a different perspective.

### 4.3. Case Study for an Enterprise Hedging against Risk

Due to the short-term and seasonal effects of air quality on enterprise operation, the design of options contracts could be based on weekly, monthly, or quarterly contracts. To demonstrate the practical application of AQI options, we chose a quarterly AQI call options contract as a case study to illustrate how enterprises can hedge against the risk caused by air pollution.

Taking a tourism company as a case study whose destination is mainly in Xi’an. If the company receives an average of 5000 passengers per month and spends about 2000 CNY person, then to avoid the risk that poor air quality in the first quarter of 2018 will reduce its revenues by reducing the number of tourists, the company could decide to buy 1500 call options for quarterly AQI at time T−t=0.8 with a strike price of 6000 CNY (assuming a strike index of 2.0, at 3000 CNY per unit). The maximum delivery index would be 2.5. According to the pricing results for AQI options in the first quarter described in Section 4.2, the company would need to pay an options premium of 331,545 CNY (=221.03 CNY/share × 1500 shares).

If poor air quality causes the number of tourists for the company to decrease by 9% below the average, this would result in a loss of 900,000 CNY (=5000 × 9% × 2000). If the AAQI at expiration is 2.3 (higher than the strike index), the company can execute the options contract. According to Equation (18), the payoff per share is 900 CNY based on *P*(*t*)=*m*[*D*(*T*) – *K*] =3000 × (2.3 – 2.0) =900. After subtracting the premium payment of 221.03 CNY per share, the actual net income is 678.97 CNY, and 1500 shares earns is 1,018,455 CNY. Since the company bought the call options, it did not lose any money, but instead gained a net profit of 118,455 CNY (1,018,455 – 900,000). When the number of tourists decreases by 10.185%, the earnings from the call options can just offset the company’s losses. If other conditions remain unchanged and the number of tourists decreases by more than 10.19%, the company will suffer losses.

If the AAQI at options expiration is 1.9 (less than the strike index), the company does not exercise the call options and only pays the options cost of 331,545 CNY. However, due to the good air quality, the increase in the number of tourists will increase revenue, which can compensate for the expenditure on the options. It was estimated that when the number of tourists increases by more than 3.315%, the income will offset the options cost. The more tourists there are, the more profits there will be.

In this example, the company avoided the risk of fluctuation of its earnings through options trading, which illustrates the powerful ability of AQI options to hedge against the operational risk caused by decreasing air quality. Through hedging, enterprises can not only avoid risks, but may even profit from this strategy.

## 5. Conclusions

Expanded O–U model using the AR-GARCH method was used to simulate the trend variations, seasonal changes, periodicity, and random perturbation of AQI. We then developed a prediction model for AQI by eliminating the effects of autocorrelation and conditional heteroscedasticity of the residuals from the AQI time series. A more realistic AAQI index was designed for use as the underlying index for the AQI options. Using Xi’an as a case study, AQI in 2018 was predicted based on parameterization of the model using the characteristics of the AQI time series from 2013 to 2017. After calculating AAQI with the predicted AQI, we priced the AQI options using an actuarial method based on the Esscher transform. To illustrate the application of AQI options in real life, we used a tourism company as a case study to explore the effect of hedging against risk using AQI options. The results of research indicate that the company could avoid the risk of fluctuation of its earnings caused by air pollution.

The case of AQI options hedging the operational risk shows that the enterprise could lock in the operational risk in advance and manage the risk through options trading, which highlights the important role of AQI options in hedging risks caused by air pollution. In addition to tourism, AQI options could also be applied to other industries that are sensitive to air quality. The duration of the AQI options contract could also be flexibly set to account for different situations, such as weekly versus monthly contracts. This paper took Xi’an city as a case study, the methodology proposed in the research is also applicable to other cities or regions.

It is worth noting that AQI only indicates the degree of air pollution, not the concentration of specific pollutants. The concentration limits differ among the six pollutants that were used to evaluate air quality. The calculation method of AQI is based on calculating the individual AQI (IAQI) for each pollutant according to the government’s concentration limits in each class for six pollutants. The maximum index for the individual pollutants was used as the AQI, and its corresponding pollutant was identified as the primary pollutant when the AQI was greater than 50. The primary pollutant therefore varied from day to day, rather than being fixed. Therefore, AQI showed high variation, and this greatly affected the prediction accuracy. In future research, it will be necessary to predict the concentrations of the six pollutants separately before determining AQI, thereby increasing the accuracy of the predictions.

## Figures and Tables

**Table 1:** Characteristics of different models.

Model	Autocorrelation Coefficient	Partial Autocorrelation Coefficient
AR (p)	trailing	Censored after a lag of p
MA (q)	Censored after a lag of q	trailing
ARMA (p,q)	trailing	trailing

Note: AR: autoregressive model; MA: moving average model; ARMA: autoregressive moving average model.

**Table 2:** Estimation of the parameters of St in Equation (4).

Parameter	*a*	*b*	*a*_0_	*a*_1_	*m*	*w*	*b*_1_	*n*
Value	133.657	−0.097	−0.057	−0.399	3.024	−0.7	2.376	1.082

**Table 3:** Parameter estimation for *C_t_*.

**Parameter**	***c***	***c*_1_**	***c*_2_**	***c*_3_**	***c*_4_**	***c*_5_**
Value	5160.802	480.067	275.108	275.892	136.895	143.781
**Parameter**	***d*_1_**	***d*_2_**	***d*_3_**	***d*_4_**	***d*_5_**	***w***
Value	−269.079	268.271	28.073	−412.647	−335.53	0.828

**Table 4:** Parameter estimation for *C_t_*.

**F-Statistic**	43.839	***p* Value**	<0.001
Obs*R-Squared	42.855	***p* Value**	<0.001

**Table 5:** The results of ARCH–LM test for conditional heteroskedasticity.

**F-Statistic**	0.725	***p* Value**	0.394
Obs*R-Squared	0.726	***p* Value**	0.395

## Appendix A. The Range and Classification Criteria of AQI

**Table A1:** The range of values and classification criteria for the air quality index (AQI).

AQI	Air Quality Level	Air Quality Situation and Color		Health Effects
0−50	1	Excellent	green	There is basically no air pollution and people can go outside normally.
51−100	2	Good	yellow	Some pollutants may have a weak impact on a small number of sensitive groups, and such groups are advised to reduce outdoor activities.
101−150	3	Mild pollution	orange	The symptoms will be slightly aggravated for susceptible groups, and healthy individuals will experience some irritation.
151−200	4	Moderate pollution	red	Symptoms will be exacerbated in susceptible populations, and the pollution may have effects on the heart and respiratory system of healthy individuals.
201−300	5	heavy pollution	purple	Patients with heart and lung disease experience significant increases in symptoms, exercise tolerance decreases, and symptoms are common even in healthy individuals.
>300	6	Serious pollution	maroon	Exercise tolerance will be reduced even for healthy individuals, and there will be obvious strong symptoms even for this group.

## Appendix B. Derivation of the Actuarial Pricing of AQI Options

For ease of calculation, assuming T−t=u, with u∈[0,T]. In a random process, after Esscher transforms of the parameter, F(x,t;h) and f(x,t;h) are the cumulative probability distribution function and probability density function, respectively. At this time, the price of call options is:

(A1) $$\begin{array}{l} C_{t}=\exp[-r(T-t)]E_{t}^{*}[P(T)]\\ \;\;\;\;\;\;\;\;\;\;\;\;\;\;=\exp[-r(u)]\{\int_{K}^{H}[(D(T)-K)f_{X}(x,T-t;h^{*})]dx+\int_{H}^{+\infty }[(H-K)f_{X}(x,T-t;h^{*})]dx\} \end{array}$$

Assuming m=ln[H/D(t)] and n=ln[K/D(t)], then:

(A2) $$\begin{array}{l} C_{t}=\exp[-r(u)]\int_{n}^{m}[(D(T)-K)f_{X}(x,T-t;h^{*})]dx+\\ \;\;\;\;\;\;\;\;\;\;\;\;\;\;\;\;\;\;\;\;\;\;\exp[-r(u)]\int_{m}^{+\infty }[(H-K)f_{X}(x,T-t;h^{*})]dx\\ \;\;\;\;\;\;\;\;\;\;\;\;\;\;=\exp[-r(u)]\{\int_{n}^{m}[(D(t)\exp(a(u))-K)f_{X}(x,T-t;h^{*})]dx\\ \;\;\;\;\;\;\;\;\;\;\;\;\;\;\;\;\;\;\;\;\;\;\;\;+\exp[-r(u)]\int_{m}^{+\infty }[(H-K)f_{X}(x,T-t;h^{*})]dx\}\\ \;\;\;\;\;\;\;\;\;\;\;\;\;\;=\;\exp[-r(u)]D(t)\int_{n}^{m}\left[\exp a(u)f(a,u;h^{*}\right)]dx-K\;\exp[-r(u)]\int_{n}^{m}f(a,u;h^{*})dx\\ \;\;\;\;\;\;\;\;\;\;\;\;\;\;\;\;\;\;\;\;\;\;+(H-K)\exp[-r(u)]\int_{m}^{+\infty }f(a,u;h^{*})dx\\ \;\;\;\;\;\;\;\;\;\;\;\;\;=\;\exp[-r(u)]D(t)\int_{n}^{m}\left[\exp a(u)f(a,u;h^{*}\right)]dx-[K\exp(-r(u))F(x,u;h^{*})]|{}_{n}^{m}\\ \;\;\;\;\;\;\;\;\;\;\;\;\;\;\;\;\;\;\;\;+(H-K)[F(x,u;h^{*})]|{}_{m}^{+\infty }\}\\ \;\;\;\;\;\;\;\;\;\;\;\;\;=D(t)\int_{n}^{m}\frac{\exp(a(u))f(a,u;h^{*})}{\exp(ru)}dx-K\exp[-r(T-t)][F(m,u;h^{*})-F(n,u;h^{*})]\\ \;\;\;\;\;\;\;\;\;\;\;\;\;\;\;\;\;\;\;\;\;\;(H-K)\;\exp[-r(T-t)][1-F(m,u;h^{*})] \end{array}$$

On this basis, we conclude that the options price for the air quality index (AQI) is as follows:

(A3) $$\begin{array}{l} C_{t}=D(t)[F(m,T-t;h^{*}+1)-F(n,T-t;h^{*}+1)]+\exp(-r(T-t))\\ \;\;\;\;\;\;\;\;\;\;\;\;\;\;\;\;\;\;\;\;\;\;[H-K\;-HF(m,T-t;h^{*})+KF(n,T-t;h^{*})] \end{array}$$

For ease of understanding, Equation (A3) can instead be expressed as:

(A4) $$\begin{array}{l} C_{t}\;=D(t)[F(m,T-t;h^{*}+1)-F(n,T-t;h^{*}+1)]\\ \;\;\;\;\;\;\;\;\;\;\;\;\;\;\;\;\;\;\;\;\;-K\exp(-r(T-t))[F(m,T-t;h^{*})-F(n,T-t;h^{*})]\\ \;\;\;\;\;\;\;\;\;\;\;\;\;\;\;\;\;\;\;\;\;+(H-K)\exp(-r(T-t))[\;1-F(m,T-t;h^{*})] \end{array}$$

## Appendix C. Pricing Formula for AQI Options under a Wiener Distribution

Since the incremental process of AAQI follows the wiener distribution, with a mean value of μu and variance $\sigma^{2}u$, the moment generating function obtained after Esscher transforms with parameter h is as follows.

(A5) $$M[z,u;h]=\exp\{[(\mu +h\sigma^{2})z+\sigma^{2}z/2]u\}.$$

According to Equation (A4) in Appendix B, the price of AQI call options under the wiener distribution can be calculated as follows:

(A6) $$\begin{array}{l} C(t)=D(t)[N(m;(\mu^{*}+\sigma^{*}{}^{2})(T-t),\sigma^{*}{}^{2}(T-t))-N(n;(\mu^{*}+\sigma^{*}{}^{2})(T-t),\sigma^{*}{}^{2}(T-t)]\\ \;\;\;\;\;\;\;\;\;\;\;\;\;\;\;\;\;\;\;\;\;\;\;\;\;+\exp(-r(T-t))[H-K-HN(m;\mu^{*}(T-t),\sigma^{*}{}^{2}(T-t))+KN(n;\mu^{*}(T-t),\sigma^{*}{}^{2}(T-t))] \end{array}$$

where * refers to a value after the transform and where

(A7) $$\mu^{*}=\frac{\mu u+h_{1}^{*}\sigma^{2}u}{T-t}=r-\sigma^{*}{}^{2}/2,\;\sigma^{*}{}^{2}=\frac{\sigma^{2}u}{T-t}.$$
